# The Prevalence of Depression and Its Potential Link to Liver Fibrosis in Patients Diagnosed With Chronic Hepatitis C Virus Infection Prior to the Initiation of Direct-Acting Antiviral Treatment

**DOI:** 10.7759/cureus.62970

**Published:** 2024-06-23

**Authors:** Andreea Florentina Stoenescu, Corneliu Petru Popescu, Simin Aysel Florescu, Geta Vancea, Emanoil Ceausu, Petre Calistru

**Affiliations:** 1 Infectious Diseases, Carol Davila University of Medicine and Pharmacy, Dr. Victor Babes Hospital of Infectious and Tropical Diseases, Bucharest, ROU; 2 Infectious Diseases, Academy of Medical Sciences, Dr. Victor Babes Hospital of Infectious and Tropical Diseases, Bucharest, ROU; 3 Infectious Diseases, Carol Davila University of Medicine and Pharmacy, Bucharest, ROU

**Keywords:** depression in chronic illness, chronic hepatitis c infection (hcv), fibrosis, depression, hepatitis c

## Abstract

Introduction

Chronic hepatitis C virus (HCV) infection is associated with various extrahepatic manifestations, including depression. This study aimed to determine the prevalence of depression in treatment-naive HCV patients and explore its potential association with liver fibrosis severity.

Methodology

A consecutive cohort of 50 treatment-naive HCV patients without coinfections was enrolled over six months. Depression was assessed using the Hamilton Depression Rating Scale (HAM-D), and the liver fibrosis stage was evaluated using Fibroscan elastography.

Results

The cohort comprised 62% females (n=31) and 38% males (n=19), with ages ranging from 27 to 76 years. HAM-D scores indicated mild depression in 78% (n=39) and moderate depression in 16% (n=8) of patients. Notably, patients with mild depression displayed varying degrees of liver fibrosis (F0, F1, and F2), while all patients with moderate depression had advanced fibrosis (F3). Based on the multiple regression model, fibrosis was a statistically significant independent predictor with an unstandardized regression coefficient (B) of 3.115 (p=0.007).

Conclusions

Our findings point to a high prevalence of depression in treatment-naive HCV patients. Interestingly, there might be a link between depression severity and the stage of liver fibrosis, with advanced fibrosis potentially associated with more severe depression.

## Introduction

Chronic hepatitis C virus (HCV) infection remains a significant public health concern due to both its hepatic and extrahepatic manifestations associated with substantial morbidity and mortality [1]. Globally, approximately 71 million individuals are infected with HCV [2]. The mechanisms by which HCV induces systemic manifestations are linked to its ability to penetrate and replicate in tissues [3], induce local and systemic inflammatory responses, and cause immune-mediated phenomena and metabolic alterations [4,5]. The extrahepatic manifestations include lymphoproliferative disorders (non-Hodgkin lymphoma, mixed cryoglobulinemia), metabolic disorders (insulin resistance, type 2 diabetes mellitus), autoimmune diseases (autoimmune thyroiditis, Sjogren's syndrome), renal disorders (membranoproliferative glomerulonephritis), cardiac disorders (atherosclerosis, ischemic heart disease), dermatological disorders, and neuropsychiatric manifestations [6].

Several studies have shown that over 50% of patients diagnosed with chronic HCV infection suffer from associated neurological or psychiatric disorders [7,8]. The most common psychiatric disorder is depression, affecting approximately one-third of individuals diagnosed with chronic HCV infection [9], with a significantly higher prevalence compared to the general population. The spectrum of depressive manifestations is broad, encompassing reduced work capacity, disease denial [10], hypochondria, insomnia or prolonged sleep periods [9], and, occasionally, suicidal ideation, aggression, and anger [11]. In some cases, strained relationships with others, decreased self-esteem, loss of interest in daily activities, and impaired concentration may also occur. Some patients may exhibit cognitive dysfunction even in the early stages of the disease [10].

Depression, along with other pathologies associated with HCV infection, has a significant negative impact on the quality of life [12,13]. Depression can be reactive to the physical and psychosocial sequelae of HCV infection. Disease awareness itself, with its negative aspects such as stigmatization and social marginalization, affecting interpersonal relationships, is a risk factor for depression [14,15]. Depression may also be associated with pre-existing comorbidities such as cardiovascular disease, diabetes mellitus, metabolic syndrome, inflammatory diseases, and sensory and motor neuropathies [15].

The mechanisms involved in depression associated with HCV infection are not fully elucidated. However, several hypotheses have been proposed, including direct neuroinvasion by HCV, alteration of neurotransmitter circuits, metabolic abnormalities, and induction of a local and systemic inflammatory response [7]. HCV can replicate in the central nervous system (CNS) due to a negative strand of RNA sequences, which is the intermediate form of genome replication [16-18]. In addition, HCV can replicate in various tissues, including bone marrow, pancreas, thyroid, spleen, and lymphoid organs, and in specific cell types such as macrophages, B lymphocytes, T lymphocytes, and endothelial cells. This widespread replication pattern can have systemic effects, potentially contributing to neuropsychiatric manifestations [3,19,20].

The viral sequences of the E1 and 5' untranslated regions (UTRs) in the CNS differ from those found in the liver [7], indicating that the virus may have an independent existence within the CNS, potentially contributing to neuropathology [8]. HCV may enter the CNS via a "Trojan horse" mechanism, using peripheral blood mononuclear cells as carriers [21]. These infected cells can cross the blood-brain barrier, introducing the virus into the CNS. Once in the CNS, HCV triggers an immune response involving immune cells, cytokines, and chemokines [18]. This inflammation can contribute to neuronal damage and dysfunction, potentially leading to neuropsychiatric symptoms. The only CNS cells known to express receptors for HCV are microvascular endothelial cells, which play a crucial role in viral entry [22]. This prospective study aimed to investigate the incidence of depression in patients with chronic HCV infection prior to direct-acting antiviral (DAA) treatment and examine the influence of the fibrosis stage on the Hamilton Depression Rating Scale (HAM-D) scores.

## Materials and methods

Study design and cohort

This study involved a prospective cohort of patients diagnosed with chronic HCV, treatment-naive (no prior treatment), recruited from the Clinical Hospital of Infectious and Tropical Diseases “Dr. Victor Babeș” in Bucharest, Romania. All participants provided written informed consent. To ensure a homogeneous study population and obtain clear results, certain criteria were applied to exclude certain patients, which were as follows: viral coinfections (patients with active viral coinfections, such as hepatitis B or HIV), individuals under the age of 18 years, patients who had previously received treatment with interferon or other antiviral medications for HCV, pregnant or breastfeeding women, and individuals unable to provide informed consent.

Data collection

We collected the data from the records of consecutive patients with chronic hepatitis C treated at the Clinical Hospital of Infectious and Tropical Diseases “Victor Babes”, Bucharest. These data relating to the personal medical history of all patients were obtained during the first evaluation visit that they needed to pass through before they could be started on interferon-free therapy. No calculation regarding data sampling was made.

The study questionnaires were administered by the infectious disease doctor, during the scheduled consultation, before the initiation of DAA therapy in a room dedicated to the study activities. Completed questionnaires were collected by the physician immediately after the patient completed them and recorded in the secure digital database. Demographic and clinical data were collected at baseline. Personal history of depression and drug use disorders were also documented.

Assessment of fibrosis stage

The stage of liver fibrosis was assessed using Fibroscan elastography in all patients before the start of the treatment. Fibroscan is a non-invasive imaging technique that measures liver stiffness, which correlates with the extent of scar tissue (fibrosis). 

Assessment of depression

HAM-D was employed in all participants. HAM-D is a self-reporting tool used to assess the severity of depressive symptoms. The original HAM-D included 21 items, but only 17 are now used in clinical practice. The excluded items are obsessive symptoms, paranoid symptoms, derealization, and diurnal mood variation, as these are relatively rare in depressive syndromes and do not accurately measure depression or its intensity. Each of the 17 HAM-D items is rated on a 0-4 scale, except for items 10 (sleep disorders) and 14 (somatic symptoms), which are rated on a 0-2 scale. The scores are interpreted as follows - HAM-D score 7-17: mild depression; HAM-D score 18-24: moderate depression; and HAM-D score 25 or higher: severe depression [23].

Ethical approval

The current study protocol received the ethical approval of the Institutional Review Board of ”Dr. Victor Babeș” Hospital with approval number 19909 effective from the 28th of December 2020.

Statistical analysis

All statistical analyses were performed using IBM SPSS Statistics 25.0 (IBM Corp., Armonk, NY), while the primary data were recorded in Microsoft Excel files. For continuous data, to test whether there are any statistically significant differences between the means of two or more independent groups, we used one-way analysis of variance (ANOVA) for normally distributed data, while the Mann-Whitney U test was used for non-normally distributed data. To assess the association between two categorical variables, Pearson's chi-squared test and Yates' correction were used for a 2 x 2 table and Fisher’s exact test was used for a table with more than 2x2 categories. Multivariate regression analysis was performed to see if there are factors independently associated with HAM-D scores when adjusted for others. A 5% cut-off level of significance was utilized for all statistical tests performed.

## Results

During the six-month study period, 50 patients diagnosed with chronic HCV were selected before starting antiviral therapy, and all of them agreed to participate. The participants were aged between 27 and 76 years with a median age of 41 years. Of them, 31 (62%) were women and 26 (52%) came from urban areas (Table 1). About half of the patients (52%) had an average level of education, while 11 (50%) had graduated from a higher educational institution.

**Table 1:** Baseline characteristics of the study cohort IQR: interquartile range

Variables	Values (n=50)
Age, years, median (IQR)	41 (35-57)
Female gender, n (%)	31 (62)
Urban residence, n (%)	26 (52)
Comorbidities, n (%)	
Hypertension	12 (24)
Diabetes mellitus	8 (16)
Thyroid diseases	6 (12)
Oncological diseases	3 (6)
Psychiatric variables	
Personal history of depression	8 (16)
Personal history of drug use	10 (20)

All patients demonstrated risky behaviors for the transmission of HCV. The presence of skin tattoos was noted in 16 (32%) of the cases and a similar proportion of patients received had blood transfusions before the diagnosis of chronic HCV. Of note, 10 (20%) patients reported injectable drug use in the past. All patients had undergone dental interventions at least once in their lifetime. The most common associated pathologies among the patients were as follows: hypertension in 12 (24%) cases, diabetes in eight (16% of cases), thyroid conditions in six (12%), and oncological disease in three (6%) cases. Psychiatric conditions (anxiety disorders) were present in eight (16%) cases.

The grades of fibrosis were evaluated using the Fibroscan test in all patients, before treatment. Fibrosis grades ranged from F0 to F4. Six patients (12%) showed a grade of fibrosis F3. F1 was present in eight (16%) patients, and 14 patients had F0. After analyzing the answers to the Hamilton questionnaire, 39 (78%) of the patients obtained a score corresponding to the category of mild depression. The score corresponding to a degree of moderate depression was registered in eight (16%) cases. Three patients had scored less than 7 points. Regarding mild levels of depression, 25 (80.64%) women registered a matching score, compared to 13 (68.42%) men. Men accounted for a higher proportion of moderate depression compared to women (21.05% vs. 12.90%).

Among the 39 patients who obtained a score corresponding to the mild depression category, five (12.82%) were known to have mental illnesses and were under medical treatment, before the diagnosis of chronic HCV. Also, four (10.25%) were known to have diabetes, two (5.12%) had arterial hypertension, and two (5.12%) patients were diagnosed with thyroid as well as malignant diseases. Multivariate regression was used to analyze if the degree of fibrosis is an independent predictor of Hamilton Score values when adjusted for other factors (Table 2). Fibrosis was the only significant factor associated with a higher HAM-D score value (t-test=3.075, p=0.007).

**Table 2:** Multivariate regression coefficients and their significance *Unstandardized regression coefficients. **Standardized regression coefficients. ***t statistic (t-test). ****Statistical significance of the test BMI: body mass index

	B*	Beta**	t***	P-value****
Age	-0.030	-0.066	-0.343	0.736
BMI	0.306	0.264	1.390	0.183
Gender	1.277	0.114	0.522	0.609
Fibrosis	3.115	0.581	3.075	0.007

## Discussion

Depression is a common comorbidity in HCV patients, leading to impaired quality of life and reduced treatment adherence. Several studies have reported the prevalence of depression in HCV patients. This study focuses on patients with chronic hepatitis C before treatment. This enabled us to analyze the association between HCV infection itself and depression, removing the potential confounding factor of treatment-induced depression. In the majority of the preceding studies, liver fibrosis has not been quantified. Quantifying fibrosis allowed us to explore a potential contributing factor to depression severity that may not have been addressed in previous studies.

Early studies, such as the one by Johnson et al. (1998), have established a link between chronic hepatitis C infection and depression. Their study found a significantly higher prevalence of depression in drug users with HCV (57.2%) compared to those without (48.2%) [24]. In our study, we observed a similar trend, with 20% of patients diagnosed with depression reporting past injectable drug use. The HAM-D scores revealed that 50% of our participants had mild depression and 40% experienced moderate depression. These findings highlight the persistence of depression in this population, emphasizing its ongoing clinical relevance. Another study by Navines et al. showed the presence of depression in 18.2% of patients diagnosed with HCV, with 6.4% of patients displaying a major degree of depression [25]. In our cohort, the prevalence of a more advanced degree of depression was much lower when compared to a milder level of depression (16% vs. 78%).

Several studies have documented a higher prevalence of depression in patients with chronic HCV compared to those with hepatitis B virus (HBV) or healthy controls. For instance, Carta et al. found a 32.6% prevalence of depression in HCV patients compared to 15.1% in HBV patients [26]. Similar findings were reported in studies conducted in the USA (29.7%) [27], Germany (25.9%) [11], and other regions. In our study, we observed a prevalence of 78% mild depression and 16% moderate depression, which aligns with these previous reports. In a meta-analysis of 130,000 HCV patients, depression was documented in 24.5%, with women having a higher risk of developing this extrahepatic manifestation. This meta-analysis classified depression as the most prevalent extrahepatic manifestation of chronic hepatitis C infection [28]. Our court had a higher proportion of women (62%). Interestingly, the majority of women (80.64%) obtained scores corresponding to the mild depression range on the Hamilton scale. This finding aligns with those of the above-mentioned meta-analysis.

The severity of liver fibrosis in chronic hepatitis C patients might be linked to depression. Studies from Poland have reported a higher prevalence of depression in patients with advanced stages of the disease. One study found depression in a significant segment (70%) of 43 patients with F4 fibrosis (a very advanced stage) [29]. Similarly, another study from Poland, involving a group of 90 patients, has reported a higher frequency of depression in patients diagnosed with cirrhosis (the final stage of fibrosis) [30]. Our study aligns with these findings, demonstrating a statistically significant association between higher fibrosis grades and increased depression scores on the Hamilton scale.

Our study focuses on demonstrating the association between depression and the stage of fibrosis. A higher stage of fibrosis also means a longer period of HCV infection, which explains the high percentage of patients with depression. Our finding of a high prevalence of depression (78%) aligns with previous research, suggesting that depression is a common comorbidity among HCV patients across various populations. There is scarce data on the level of depression in HCV-infected patients from Romania. Also, studies in the current literature do not use a depression quantification tool. Our study contributes to this field by investigating the association between depression and fibrosis severity in a specific population of HCV patients. Despite the study's relatively small sample size, our findings related to patient characteristics and degree of fibrosis align with findings from other research. We employed the Hamilton scale, a validated tool for measuring depression severity, enabling us to provide valuable insights into the spectrum of depression within the HCV population.

We acknowledge that our current data cannot definitively establish a causality between depression and fibrosis severity in HCV patients. Our study delves deeper into this complex interplay by exploring the possibility of a bidirectional relationship, where depression may also exacerbate fibrosis progression. It employs a robust methodological approach, utilizing validated measures of both fibrosis severity and depression symptoms. This methodological rigor strengthens the credibility of our findings and enhances the generalizability of our results.

Early referral to healthcare professionals, such as psychologists or psychiatrists, could aid in providing improved management and care in this population. Additionally, lifestyle modifications could be explored to manage depressive symptoms. These modifications might include professional support for drug withdrawal, control of comorbidities like hypertension or diabetes, and potentially other interventions. The limited research on this topic highlights the need for further studies on the neuropsychiatric effects of HCV infection. Gaining a better understanding of depression in this population is crucial. The Hamilton scale used in this study appears to be a valuable tool for such investigations.

This study has a few limitations. We assessed the mental status of our cohort by relying solely on self-administered questionnaires, rather than structured clinical interviews conducted by a qualified mental health professional. This shortcoming, along with the relatively small sample size, may affect the generalization of findings to the broader HCV population.

## Conclusions

Our study enhances the evidence of a high prevalence of depression among HCV patients, particularly mild depression (78%). This highlights the crucial need for integrating depression screening and early intervention into routine HCV care. By addressing both conditions concurrently, we can significantly improve patients' quality of life and potentially enhance their treatment outcomes. Furthermore, the bidirectional relationship between depression and HCV underscores the importance of comprehensive healthcare approaches. Clinicians treating patients with neuropsychiatric disorders, including depression, should consider screening them for HCV infection, and vice versa.
